# Functional and Structural Outcomes of Photobiomodulation Therapy in Dry Age-Related Macular Degeneration: A Single-Center Experience

**DOI:** 10.3390/jcm15114007

**Published:** 2026-05-22

**Authors:** Sefik Can Ipek, Ceren Durmaz Engin, Ezgi Karatas, Cem Yildirim, Andrzej Grzybowski

**Affiliations:** 1 Independent Researcher, 20010 Denizli, Türkiye; sefikcanipek@gmail.com (S.C.I.); profdrcemyildirim@gmail.com (C.Y.); 2 Department of Ophthalmology, Democracy University, 35140 Izmir, Türkiye; ceren.engin@idu.edu.tr; 3 Department of Ophthalmology, Ibrahim Cecen University, 04100 Agri, Türkiye; ezkaratas@agri.edu.tr; 4 Department of Ophthalmology, University of Warmia and Mazury, 10-719 Olsztyn, Poland; 5 Institute for Research in Ophthalmology, 60-806 Poznan, Poland

**Keywords:** dry age-related macular degeneration, optical coherence tomography, photobiomodulation, retinal biomarkers

## Abstract

**Highlights:**

**What are the main findings?**

1. Photobiomodulation therapy was associated with a significant short-term improvement in visual acuity in eyes with dry age-related macular degeneration.
2. Conventional OCT-based structural parameters, including central retinal thickness, macular volume, and outer retinal band integrity, did not show significant post-treatment change, while baseline visual acuity and external limiting membrane integrity predicted visual gain.

**What are the implications of the main findings?**

1. Photobiomodulation may provide functional benefit in selected patients with dry age-related macular degeneration even in the absence of measurable anatomical recovery on routine OCT.
2. Baseline functional reserve and preserved outer retinal viability may help identify patients more likely to benefit from treatment and guide future prospective studies.

**Abstract:**

**Purpose:** To evaluate the anatomical and functional efficacy of photobiomodulation (PBM) therapy in patients with dry age-related macular degeneration (AMD) and to investigate structural predictors of visual response. **Methods:** This retrospective study included 47 eyes of 30 patients with dry AMD treated with PBM. Best-corrected visual acuity (BCVA) was recorded as Early Treatment Diabetic Retinopathy Study (ETDRS) letter score, and visual change (ΔBCVA) was calculated. Spectral-domain optical coherence tomography parameters—central retinal thickness (CRT), central macular volume (ETDRS 9-subfield central zone), and photoreceptor layer integrity (external limiting membrane [ELM], ellipsoid zone [EZ], and interdigitation zone [IZ])—were assessed pre- and post-treatment. Age-Related Eye Disease Study (AREDS) stage was graded per eye. Because both eyes from some patients were included, generalized estimating equation (GEE) models with patient-level clustering were used to account for inter-eye correlation. Effect estimates were reported as unstandardized coefficients with 95% confidence intervals. **Results:** Visual acuity improved following PBM therapy, with mean ETDRS letter scores increasing from 75.0 ± 14.1 to 78.0 ± 12.1 letters. In the GEE model accounting for patient-level clustering, the estimated mean gain was 2.97 ETDRS letters (95% CI: 1.15 to 4.79; *p* = 0.001). Mean CRT showed no significant change following PBM therapy (210.32 ± 48.61 µm vs. 211.23 ± 50.27 µm; GEE estimate: +0.91 µm; 95% CI: −5.52 to 7.35; *p* = 0.780). Central macular volume likewise remained stable (0.1913 ± 0.030 vs. 0.1919 ± 0.033 mm^3^; GEE estimate: +0.0006 mm^3^; 95% CI: −0.0054 to 0.0067; *p* = 0.836). Photoreceptor layer integrity demonstrated limited structural change, with no significant time effect for EZ or IZ integrity in binary GEE models and no observed pre–post change in ELM integrity. In multivariable GEE analysis, baseline BCVA (*p* < 0.001), ELM integrity (*p* < 0.001) and central macular volume (*p* = 0.041) were associated with change in ETDRS letter score, whereas AREDS category, EZ integrity, and IZ integrity were not. **Conclusions:** PBM therapy demonstrated limited short-term anatomical change but variable functional outcomes in dry AMD. Baseline BCVA emerged as the primary determinant of visual response, suggesting that treatment benefit may be influenced predominantly by pre-treatment functional reserve.

## 1. Introduction

Age-related macular degeneration (AMD) is a leading cause of irreversible vision loss among individuals older than 50 years worldwide and represents a major public health challenge in aging populations. The disease primarily affects the macula and leads to progressive central vision impairment that significantly reduces visual function and quality of life [1]. The non-neovascular (dry) form accounts for approximately 85–90% of AMD cases and is characterized by drusen accumulation, retinal pigment epithelium (RPE) dysfunction, and progressive photoreceptor degeneration [2]. Despite its high prevalence, effective therapeutic options for dry AMD remain limited, and current management strategies largely rely on risk factor modification and antioxidant supplementation based on the Age-Related Eye Disease Study (AREDS) recommendations [3]. The pathogenesis of dry AMD is complex and multifactorial, involving oxidative stress, mitochondrial dysfunction, impaired cellular metabolism, and chronic inflammation within the RPE–photoreceptor complex [4]. Increasing evidence suggests that mitochondrial dysfunction plays a central role in retinal aging and degeneration by reducing cellular energy production and increasing oxidative damage in retinal tissues [5]. These mechanisms have stimulated growing interest in therapeutic approaches targeting mitochondrial bioenergetics as potential disease-modifying strategies in AMD.

Photobiomodulation (PBM) therapy has emerged as a promising non-invasive approach designed to improve cellular metabolism and mitochondrial function. PBM utilizes low-level visible or near-infrared light that is absorbed by mitochondrial chromophores, particularly cytochrome c oxidase, resulting in enhanced mitochondrial respiration, increased adenosine triphosphate production, and reduced oxidative stress and inflammatory signaling [6]. Experimental studies have demonstrated that PBM can improve mitochondrial function, reduce inflammatory responses, and promote photoreceptor survival in retinal degeneration models [7]. In recent years, clinical studies have investigated the potential benefits of PBM therapy in patients with dry AMD. Early clinical reports suggested improvements in visual acuity and contrast sensitivity following PBM treatment [8]. More recently, the LIGHTSITE clinical trials evaluating multi-wavelength PBM systems reported improvements in visual acuity and potential slowing of disease progression in selected patients with intermediate dry AMD [9,10,11]. However, clinical outcomes remain heterogeneous, and the structural determinants of functional improvement after PBM therapy are still not fully understood.

Spectral-domain optical coherence tomography (SD-OCT) allows high-resolution visualization of retinal microstructure and enables detailed assessment of outer retinal layers, including the external limiting membrane (ELM), ellipsoid zone (EZ), and interdigitation zone (IZ). Integrity of these layers is strongly associated with photoreceptor health and visual function in several retinal diseases [12]. Identifying structural biomarkers that predict treatment response may therefore provide valuable insight into the mechanisms underlying PBM-associated functional improvement and assist in optimizing patient selection.

The present study aimed to evaluate the anatomical and functional outcomes of PBM therapy in patients with dry AMD in a real-world clinical setting. In addition, we investigated potential structural predictors of visual response using SD-OCT-derived parameters, including CRT, macular volume, and photoreceptor layer integrity.

## 2. Materials and Methods

### 2.1. Study Design and Population

This retrospective, observational, single-center study evaluated patients diagnosed with dry AMD who underwent photobiomodulation PBM therapy between January 2024 and January 2026. The study was conducted at a private eye hospital and adhered to the tenets of the Declaration of Helsinki. Institutional ethics committee approval was obtained from Agri Ibrahim Cecen University local ethics committee (Approval number: 217, Approval Date: 1 April 2026) Patient consent for study was waived due to retrospective design, but all patients had informed consent before PBM treatment.

A literature-based minimum sample size calculation was performed. According to the previous studies, assuming a 4-letter improvement in best-corrected visual acuity (BCVA), a standard deviation of paired differences of 5 letters, a two-sided alpha of 0.05, and 80% power, at least 25 patients are required [10,13,14].

Patients were eligible for inclusion if they were diagnosed with non-neovascular AMD characterized by the presence of drusen and/or RPE alterations consistent with dry AMD. All included eyes were required to have available baseline and post-treatment SD-OCT imaging, to have completed at least one full course of PBM therapy, and to have a minimum follow-up duration of 2 months. In addition, only eyes with sufficient image quality allowing reliable quantitative measurements and qualitative outer retinal layer assessment were included in the analysis. Eyes were excluded if neovascular AMD (choroidal neovascularization) was present or if there was any history of intravitreal anti-vascular endothelial growth factor (anti-VEGF) therapy. Eyes with central geographic atrophy involving the fovea were also excluded. Additional exclusion criteria comprised coexisting retinal pathologies such as diabetic retinopathy, retinal vein occlusion, or macular hole, prior vitreoretinal surgery, media opacities affecting image acquisition quality, and incomplete clinical or imaging records. Furthermore, electrophysiological assessments were not analyzed because they were not obtained in all patients.

### 2.2. PBM Treatment Protocol

All patients received PBM therapy using a multi-wavelength light delivery system (Valeda^®^ Light Delivery System, LumiThera Inc., Poulsbo, WA, USA). Treatment was administered according to the manufacturer’s recommended protocol, consisting of 9 sessions per treatment course, typically delivered 3 times per week over 3–5 weeks. Each session delivered light at wavelengths of 590 nm, 660 nm, and 850 nm. The treatment time per session was 250 s (approximately 4.2 min) per eye, with 590 nm and 850 nm applied in two 35 s phases each and 660 nm applied in two 90 s phases. The number of completed treatment courses was recorded for each patient.

### 2.3. Ophthalmic Examination

All patients underwent comprehensive ophthalmologic evaluation at baseline and follow-up visits. The examination protocol included BCVA measurement, slit-lamp biomicroscopy, dilated fundus examination, and SD-OCT imaging. BCVA was assessed using Early Treatment Diabetic Retinopathy Study (ETDRS) charts at a testing distance of 4 m. ΔBCVA was defined as post-treatment BCVA minus baseline BCVA, such that positive values indicate visual improvement. Comparisons were made between the initial and last examination results.

### 2.4. OCT Acquisition and Analysis

Macular imaging was performed using SD-OCT (Mirante SLO/OCT, NIDEK Co., Ltd., Gamagori, Japan), using the device’s macular map scan protocol. Quantitative structural parameters were automatically generated by the device software. Central retinal thickness, expressed in micrometers, and central macular volume derived from the ETDRS 9-subfield map (central 1 mm zone) were recorded for each eye. Direct drusen volume quantification was not available with the imaging platform used; therefore, central macular volume was evaluated as an indirect and limited structural parameter rather than a direct measure of drusen burden. Outer retinal microstructural integrity was qualitatively evaluated on high-resolution B-scan OCT images. Assessments were performed independently by two masked retina specialists. The integrity of the ELM, EZ, and IZ was analyzed. Each layer was graded dichotomously as either intact or disrupted. Intergrader agreement for ELM, EZ, and IZ integrity grading, classified as intact or disrupted, was assessed using Cohen’s kappa statistics.

### 2.5. AREDS Classification

Each eye was categorized according to the AREDS classification system based on multimodal structural evaluation, including dilated fundus examination, color fundus imaging, and OCT findings [2,13]. Eyes were classified as Category 2, 3, or 4 according to drusen burden, pigmentary abnormalities, and the presence of advanced AMD features. Category 2 represented early AMD with small or limited intermediate drusen and/or mild pigmentary changes. Category 3 represented intermediate AMD with extensive intermediate drusen, at least one large druse, and/or noncentral geographic atrophy. Category 4 represented advanced AMD features, including geographic atrophy; however, eyes with central foveal involvement were excluded. Thus, Category 4 eyes in this study represented advanced AMD without foveal center involvement.

### 2.6. Statistical Analysis

Statistical analyses were conducted using IBM SPSS Statistics for Windows, version 25.0 (IBM Corp., Armonk, NY, USA). Data distribution normality was assessed using the Kolmogorov–Smirnov and Shapiro–Wilk tests. Descriptive statistics were presented as mean ± standard deviation for continuous variables and as frequencies and percentages for categorical variables.

Because both eyes from the same patient were included in a substantial proportion of cases, inter-eye correlation was accounted for using generalized estimating equations (GEEs) with patient-level clustering, Gaussian distribution, identity link function, exchangeable working correlation structure, and robust standard errors. Effect estimates were reported as unstandardized coefficients with 95% confidence intervals.

The primary pre–post comparison of visual acuity was performed using a GEE model with ETDRS letter score as the dependent variable and time point, baseline versus post-treatment, as the main independent variable. Structural OCT outcomes were also reanalyzed using GEE models with patient-level clustering. Continuous OCT parameters, including CRT and central macular volume, were analyzed using Gaussian GEE models with identity link function, whereas dichotomous ELM, EZ, and IZ integrity outcomes were analyzed using binary GEE models with logit link function when estimation was possible. Because ELM integrity showed no discordant pre–post change, it was summarized descriptively. Associations between AREDS category and baseline photoreceptor layer disruption were summarized descriptively and evaluated using Chi-square or Fisher’s exact tests, as appropriate.

Factors associated with visual acuity change were evaluated using change in ETDRS letter score as the dependent variable. Univariable and multivariable GEE models with patient-level clustering were fitted. The multivariable model included baseline visual acuity, AREDS category, and baseline ELM, EZ, and IZ integrity. Because baseline visual acuity is mathematically related to visual acuity change, this model was interpreted as a sensitivity analysis and evaluated cautiously due to potential regression-to-the-mean bias.

Given the absence of a sham-treated or untreated control group, all findings were interpreted as within-subject associations rather than causal evidence of treatment efficacy. A *p*-value <0.05 was considered statistically significant.

## 3. Results

**Figure 1:**
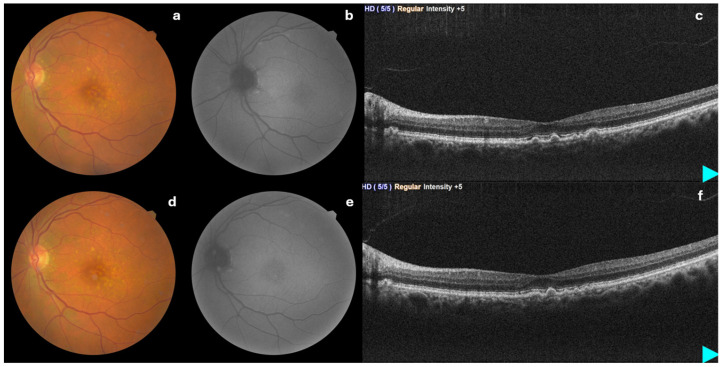
Representative pre-treatment and post-treatment multimodal retinal images of a 57-year-old female patient with dry age-related macular degeneration following photobiomodulation therapy. The upper row shows pre-treatment images, and the lower row shows post-treatment images. Color fundus photography ((**a**), pre-treatment; (**d**), post-treatment) shows multiple yellowish drusen and mottled pigmentary changes at the macula. Fundus autofluorescence ((**b**), pre-treatment; (**e**), post-treatment) shows heterogeneous autofluorescence alterations, including central irregular hypoautofluorescent and hyperautofluorescent areas associated with drusen-related RPE changes in the macular region. OCT images ((**c**), pre-treatment; (**f**), post-treatment) show drusenoid RPE elevations and irregularities of the RPE–Bruch’s membrane complex in the macular region.

A total of 30 patients were included. The mean age was 72.67 ± 9.14 years (range: 54–89). There were 7 males (23.3%) and 23 females (76.7%). Bilateral involvement was present in 56.7% of patients. Diabetes mellitus and hypertension were observed in 23.3% and 50.0% of patients, respectively. The mean number of treatment courses was 1.30 ± 0.75 (range: 1–4), and the mean follow-up duration was 6.90 ± 4.60 months (range: 2–20). Representative pre-treatment and post-treatment multimodal images of a 57-year-old female patient are shown in Figure 1.

Visual acuity showed a modest improvement following PBM therapy. Mean ETDRS letter score increased from 75.0 ± 14.1 at baseline to 78.0 ± 12.1 after treatment, corresponding to a mean gain of 3.0 ± 5.7 ETDRS letters. In the GEE model accounting for patient-level clustering, the estimated mean gain was 2.97 ETDRS letters (95% CI: 1.15 to 4.79; *p* = 0.001). Change in ETDRS letter score did not differ significantly across AREDS categories (*p* = 0.154).

Intergrader agreement was excellent for ELM (κ = 0.846), substantial for EZ (κ = 0.777), and excellent for IZ (κ = 0.911), indicating overall substantial-to-excellent agreement. Photoreceptor layer disruption was significantly associated with higher AREDS categories. EZ damage was observed in 6.3% of Category 2 eyes, 4.2% of Category 3 eyes, and 71.4% of Category 4 eyes (*p* < 0.001). IZ disruption was present in 18.8%, 62.5%, and 100% of eyes across Categories 2, 3, and 4, respectively (*p* = 0.001). ELM disruption was detected in 6.3%, 4.2%, and 42.9% of eyes across the same categories (*p* = 0.011). The distribution of these findings according to AREDS categories is presented in Table 1.

No significant structural recovery was observed following treatment in GEE analyses accounting for patient-level clustering. CRT and central macular volume remained stable after PBM therapy. The estimated post-treatment change was +0.91 µm for CRT (95% CI: −5.52 to 7.35; *p* = 0.780) and +0.0006 mm^3^ for central macular volume (95% CI: −0.0054 to 0.0067; *p* = 0.836). Intact ELM was present in 42 eyes (89.4%) both at baseline and after PBM therapy, while disruption persisted in 5 eyes; because no discordant pre–post ELM changes were observed, a binary GEE time-effect estimate could not be reliably calculated. Intact EZ was observed in 40 eyes (85.1%) at baseline and 38 eyes (80.9%) after PBM therapy; binary GEE analysis showed no significant change (OR: 0.74; 95% CI: 0.50 to 1.11; *p* = 0.148). Similarly, intact IZ was present in 22 eyes (46.8%) at baseline and 20 eyes (42.6%) after PBM therapy, with no significant time effect in binary GEE analysis (OR: 0.84; 95% CI: 0.67 to 1.06; *p* = 0.147).

In univariable GEE analyses accounting for patient-level clustering, baseline BCVA was significantly associated with change in ETDRS letter score, as shown in Table 2. Higher baseline BCVA was associated with a higher subsequent visual gain coefficient: 0.24 ETDRS letters per 1-letter increase in baseline BCVA (*p* < 0.001). Baseline macular volume was also significantly associated with visual acuity change coefficient: −0.74 ETDRS letters per 0.01 mm^3^ increase (*p* = 0.004). No significant associations were observed for age, sex, diabetes mellitus, AREDS category, baseline CRT, ELM integrity, EZ integrity, IZ integrity, number of treatment courses, or follow-up duration.

Multivariable GEE analysis was performed to evaluate factors associated with change in ETDRS letter score while accounting for patient-level clustering. In this model, baseline visual acuity was significantly associated with visual acuity change. Each 1-letter increase in baseline visual acuity was associated with a 0.22-letter greater change in ETDRS score (95% CI: 0.09 to 0.35; *p* < 0.001). Baseline ELM integrity was also significantly associated with greater visual acuity gain (coefficient: +0.86 ETDRS letters; 95% CI: 0.41 to 1.31; *p* < 0.001). In contrast, higher baseline macular volume was significantly associated with a smaller visual acuity gain; each 0.01 mm^3^ increase in baseline macular volume was associated with a 0.49-letter lower change in ETDRS score (95% CI: −0.96 to −0.02; *p* = 0.041). AREDS category, EZ integrity, IZ integrity, and baseline CRT were not significantly associated with visual acuity change. The results of the multivariable GEE analysis are presented in Table 3.

## 4. Discussion

Photobiomodulation has emerged as a promising non-invasive therapy for dry AMD, targeting mitochondrial dysfunction and oxidative stress. In our study, PBM therapy was associated with a modest short-term improvement in visual acuity without measurable short-term anatomical changes on OCT, while baseline visual acuity, ELM integrity and macular volume were associated with visual response in multivariable GEE analysis accounting for patient-level clustering.

### 4.1. Visual Acuity Change

In our cohort, BCVA improved significantly after PBM, increasing from 75.0 ± 14.1 to 78.0 ± 12.1 ETDRS letters, corresponding to an average gain of approximately 3 letters, but the magnitude of improvement did not differ significantly across AREDS categories. This pattern is broadly consistent with the visual improvement reported in the LIGHTSITE program and other PBM studies, although reported determinants of response differ across studies. In LIGHTSITE I, greater visual response was mainly observed in eyes with AREDS category 3 disease or category 4 disease with foveolar sparing, suggesting that preserved central anatomy may support stronger BCVA improvement after PBM [10]. LIGHTSITE II and III reported favorable functional outcomes in PBM-treated eyes, but did not provide formal baseline responder models; however, LIGHTSITE III showed an association between higher BCVA and lower macular drusen volume, suggesting that functional benefit may be linked to a more favorable structural profile [9,11].

Outside the LIGHTSITE trials, Merry et al. [14] reported that eyes with intermediate baseline BCVA (70–89 ETDRS letters) were more likely to gain >5 letters, suggesting a potential ceiling effect in eyes with better initial vision. More recently, PBM4AMD showed that greater BCVA improvement was associated with lower baseline BCVA, higher baseline contrast sensitivity, and lower triglyceride levels, while contrast sensitivity improvement was linked to higher baseline drusen volume [15]. In contrast, although our cohort showed a statistically significant visual gain, BCVA change was not associated with AREDS category. This may reflect the relatively small sample size, limited number of advanced eyes, and generally preserved baseline visual acuity in our series. In our multivariable GEE analysis, baseline visual acuity was associated with change in ETDRS letter score, a finding that differs from the PBM4AMD study. However, because baseline visual acuity is mathematically related to change-score outcomes, this finding should not be interpreted as definitive evidence that better baseline vision independently predicts treatment response. Rather, it should be considered a sensitivity finding that may reflect baseline functional reserve, regression-to-the-mean effects, or both.

Several studies, including ours, suggest visual improvement after PBM; however, the evidence remains heterogeneous. A 670 nm pilot study in intermediate AMD found no significant functional or structural improvement at 1, 3, 6, or 12 months [16]. Similarly, a recent meta-analysis of randomized trials reported a modest pooled BCVA gain of 1.76 letters, indicating that PBM-related visual benefit may be statistically significant but often limited in magnitude [17].

Although the mean BCVA improvement of approximately 3 ETDRS letters may appear modest, its clinical relevance should be interpreted in the context of chronic, progressive diseases such as AMD, where even small functional gains or stabilization may be meaningful. In several clinical trials, including studies in retinal diseases such as neovascular AMD, relatively small changes in ETDRS letter scores have been considered relevant, particularly when associated with disease stabilization or reduced risk of further vision loss. Therefore, the observed improvement in our cohort, although limited in magnitude, may still reflect a clinically relevant functional benefit in selected patients. Nevertheless, this finding should be interpreted cautiously given the retrospective design and absence of a control group.

Taken together, these findings suggest that PBM therapy may provide functional improvement in selected patients with dry AMD, although the magnitude and durability of the effect may vary depending on disease stage, difference in age distribution and follow-up duration.

### 4.2. Retinal Thickness Changes

In our cohort, neither CRT nor central macular volume changed significantly after PBM treatment. These findings are broadly consistent with previous PBM studies in dry AMD, in which functional improvement was not necessarily accompanied by measurable changes in conventional OCT thickness parameters. In a study by Merry et al. [14], visual and drusen-related improvements were reported, whereas overall CRT and retinal volume remained stable. Likewise, LIGHTSITE I did not demonstrate a significant change in retinal volume or CRT despite functional improvement and a reduction in drusen volume in PBM-treated eyes [10].

A key difference is that most prior studies evaluated structural response mainly using drusen-based parameters rather than conventional OCT thickness metrics. In LIGHTSITE II, drusen volume and central subfield drusen thickness increased in sham-treated eyes but remained stable or slightly decreased in PBM-treated eyes, although these differences were not statistically significant [11]. Similarly, in LIGHTSITE III, macular drusen volume remained relatively stable in PBM-treated eyes but increased in sham-treated eyes [9].

Because drusen volume analysis was not available in our study, we relied on CRT and central macular volume as conventional OCT-derived structural parameters. However, central macular volume should not be considered equivalent to drusen volume, and it may be less sensitive for detecting PBM-related anatomical changes in dry AMD. Although automated segmentation software for drusen volume measurement is available for platforms such as Zeiss and Heidelberg, there is currently no validated or approved program for the Mirante device used in our study. Therefore, drusen volume could not be quantified. This is relevant because the existing literature suggests that PBM-related anatomical effects, if present, may be better captured by drusen-specific measurements rather than by global retinal thickness or volume metrics.

### 4.3. Structural Alterations in the Outer Retina

Our study demonstrated a strong association between photoreceptor layer disruption and higher AREDS categories, particularly for EZ and IZ integrity. This finding aligns with the known pathophysiology of AMD, in which progressive degeneration of photoreceptors and RPE leads to structural disruption of the outer retinal layers [18].

In our study, no significant structural recovery was observed in the outer retinal bands after PBM therapy. ELM integrity remained unchanged, with intact ELM present in 42 eyes both before and after treatment, whereas EZ and IZ showed slight worsening, although neither change was statistically significant. These findings are broadly consistent with the existing PBM literature in dry AMD. In the LIGHTSITE I and TORPA studies, outer retinal bands such as the ELM, EZ, and IZ were assessed as part of the OCT grading protocol, but clear quantitative restoration of these layers was not reported as a major anatomical outcome [10,14]. Instead, the structural signal of PBM was mainly reflected by drusen-related parameters, while representative images showed no new disruption of the photoreceptor layers despite drusen regression. Similarly, in later reports, including real-world Valeda series and recent safety analyses, no new onset EZ/IZ irregularities or other signs of phototoxic outer retinal damage were observed, but consistent regeneration of the photoreceptor bands was also not demonstrated [11,19].

An important finding in our cohort was that baseline ELM integrity was associated with greater visual acuity gain in the multivariable GEE model, whereas EZ and IZ integrity were not. The finding that ELM integrity was not significant in univariable analysis but emerged as an independent predictor in the multivariable model may reflect adjustment for baseline functional status, confounding, or shared variance among related outer retinal parameters. This may be clinically meaningful, because the ELM may represent a more stable marker of preserved photoreceptor-Müller cell interface and residual outer retinal viability than EZ or IZ alone [20,21].

Although previous PBM studies did not formally identify ELM as an independent predictor, their overall results point in the same direction: better visual response was generally observed in eyes with earlier-stage disease, better baseline vision, and less central tissue loss [9,10,14]. Therefore, our data suggests that preserved baseline ELM integrity may be associated with greater functional change after PBM, even when PBM does not produce visible restoration of EZ, IZ, or ELM on OCT, preserved baseline ELM integrity may still indicate a retina with sufficient functional reserve to benefit from treatment. In this context, the absence of overt outer retinal band recovery does not necessarily contradict the functional gain observed after PBM, but rather suggests that visual improvement may precede, exceed, or occur independently of detectable morphological repair on conventional OCT. Given the retrospective design, limited sample size, and absence of a sham-treated or untreated control group, ELM integrity should be regarded as a potential structural marker associated with visual response rather than a confirmed independent predictor of treatment efficacy.

Experimental studies may support this interpretation. PBM has been shown to enhance mitochondrial activity within photoreceptors and RPE cells by stimulating cytochrome-c oxidase, a key enzyme in the mitochondrial respiratory chain [22]. Activation of this pathway may increase ATP production, reduce reactive oxygen species, and promote cellular survival [23]. Therefore, observed functional changes after PBM may depend more on intracellular physiological and metabolic modulation than on detectable anatomical restoration, allowing photoreceptor performance to improve even in the absence of overt structural regeneration.

The present study has several strengths. First, it represents a single-center experience with a relatively large patient cohort compared with many previous PBM studies, which strengthens the internal consistency of the findings. Second, the simultaneous evaluation of both anatomical and functional outcomes provides a comprehensive assessment of treatment response, allowing a more complete interpretation of the observed clinical findings after PBM therapy. Finally, the study reflects real-world clinical practice, offering valuable real-world evidence regarding the short-term anatomical and functional outcomes associated with PBM in dry AMD.

### 4.4. Limitations

Several limitations should also be acknowledged. The sample size, although relatively substantial for a single-center study, remains limited and may restrict statistical power. In addition, the follow-up duration was not uniform and relatively short in some patients, which may limit the evaluation of long-term visual outcomes, disease progression, and durability of the observed changes after PBM. A major limitation is the heterogeneity in PBM exposure and follow-up duration. The number of treatment courses ranged from 1 to 4, and follow-up duration ranged from 2 to 20 months, which may have influenced the assessment of treatment response, disease progression, and durability of PBM-related effects. Given the variability in follow-up duration and treatment session number, we additionally assessed their relationship with visual acuity improvement to account for these potential sources of heterogeneity. Neither parameter showed a significant association with visual outcome, suggesting that the observed visual change was not significantly associated with differences in follow-up time or treatment frequency in this cohort. Another limitation is the absence of drusen-related OCT measurements, such as drusen area, volume, or thickness, due to technical limitations of the imaging platform. These parameters have been among the most informative structural outcomes in previous PBM studies and may have provided a more sensitive assessment of anatomical change. Finally, electrophysiological assessments were not analyzed because they were not obtained in all patients. Future studies with larger multicenter cohorts and longer follow-up periods will be important to further clarify the long-term efficacy and clinical role of PBM therapy.

## 5. Conclusions

The results of our study contribute to the growing body of evidence suggesting that PBM therapy may be associated with short-term functional improvement in patients with dry AMD. In our cohort, PBM was associated with a significant improvement in visual acuity, whereas no significant changes were observed in conventional OCT-based structural parameters, including CRT, macular volume, and outer retinal band integrity. These findings support the concept that the observed functional changes after PBM may be mediated primarily through metabolic and cellular mechanisms rather than through rapid anatomical changes detectable by routine OCT imaging.

Our findings also highlight the importance of baseline retinal function and outer retinal integrity in relation to visual response. Better baseline BCVA and preserved ELM integrity were associated with greater visual acuity gain, suggesting that retinal functional reserve and outer retinal viability may play a role in identifying patients with greater observed visual change after PBM. At the same time, the absence of an association between visual gain and AREDS category indicates that conventional clinical staging alone may not fully capture functional responsiveness.

Taken together, these results suggest that PBM was associated with modest short-term visual improvement in selected patients with dry AMD, even in the absence of measurable structural recovery. However, causal conclusions regarding treatment efficacy cannot be drawn because of the retrospective design and absence of a sham-treated or untreated control group. The heterogeneity of the current literature and the limitations of our study, including the lack of drusen-specific OCT biomarkers and variable follow-up duration, indicate that further prospective, multicenter, controlled studies with larger cohorts and longer observation periods are needed to better define the structural correlates, predictive factors, and long-term clinical relevance of PBM therapy in dry AMD.

## Figures and Tables

**Table 1:** Distribution of EZ, IZ, and ELM disruption according to AREDS category.

AREDS	EZ Disruption (n)	EZ Intact (n)	Disruption %
Cat 2	1	15	6.3%
Cat 3	1	23	4.2%
Cat 4	5	2	71.4%
AREDS	IZ disruption (n)	IZ intact (n)	Disruption %
Cat 2	3	13	18.8%
Cat 3	15	9	62.5%
Cat 4	7	0	100%
AREDS	ELM disruption (n)	ELM intact (n)	Disruption %
Cat 2	1	15	6.3%
Cat 3	1	23	4.2%
Cat 4	3	4	42.9%

**Table 2:** Univariable GEE analysis of factors associated with change in ETDRS letter score, accounting for patient-level clustering.

Variable	Coefficient, ETDRS Letters	95% CI	*p*-Value
Age, per year	+0.02	−0.21 to 0.26	0.859
Sex, female vs. male	+2.25	−2.36 to 6.87	0.339
Diabetes mellitus	+2.27	−1.54 to 6.08	0.243
Hypertension	+3.69	0.12 to 7.26	0.053
**Baseline BCVA, per 1 ETDRS letter**	**+0.24**	**0.12 to 0.36**	**<0.001**
AREDS category, per category increase	+2.38	−0.57 to 5.33	0.113
Baseline CRT, per 10 µm	−0.16	−0.59 to 0.27	0.469
**Baseline MV, per 0.01 mm^3^**	**−0.74**	**−1.25 to −0.23**	**0.004**
ELM integrity, intact vs. disrupted	+0.35	−2.02 to 2.72	0.771
EZ integrity, intact vs. disrupted	−2.08	−6.27 to 2.11	0.331
IZ integrity, intact vs. disrupted	−1.61	−4.77 to 1.55	0.318
Number of treatment courses	+0.94	−2.51 to 4.38	0.595
Follow-up duration, months	+0.01	−0.48 to 0.49	0.978

AREDS, Age-Related Eye Disease Study; BCVA, best-corrected visual acuity; CRT, central retinal thickness; DM, diabetes mellitus; ELM, external limiting membrane; EZ, ellipsoid zone; GEE, Generalized Estimating Equations; HT, hypertension; IZ, interdigitation zone; MV, macular volume. Bold formatting is used to indicate statistically significant variables.

**Table 3:** Multivariable GEE analysis of factors associated with change in ETDRS letter score, accounting for patient-level clustering.

Variable	Coefficient, ETDRS Letters	95% CI	*p*-Value
**Baseline VA, per 1 ETDRS letter**	**+0.22**	**0.09 to 0.35**	**<0.001**
AREDS category 3 vs. 2	+0.57	−2.59 to 3.74	0.722
AREDS category 4 vs. 2	+4.91	−3.17 to 12.99	0.234
**ELM integrity, intact vs. disrupted**	**+0.86**	**0.41 to 1.31**	**<0.001**
EZ integrity, intact vs. disrupted	−0.58	−1.47 to 0.30	0.197
IZ integrity, intact vs. disrupted	−0.33	−3.30 to 2.64	0.825
Baseline CRT, per 10 µm	+0.31	−0.09 to 0.71	0.132
**Baseline MV, per 0.01 mm^3^**	**−0.49**	**−0.96 to −0.02**	**0.041**

AREDS, Age-Related Eye Disease Study; BCVA, best-corrected visual acuity; CRT, central retinal thickness; DM, diabetes mellitus; ELM, external limiting membrane; EZ, ellipsoid zone; GEE, Generalized Estimating Equations; HT, hypertension; IZ, interdigitation zone; MV, macular volume; VA, visual acuity. Baseline VA was included because of clinical relevance; however, this model was interpreted as a sensitivity analysis because baseline VA is mathematically related to change in ETDRS letter score. Bold formatting is used to indicate statistically significant variables.

## Data Availability

Data are available upon reasonable request from the corresponding author.

## Abbreviations

The following abbreviations are used in this manuscript:

AMD	Age-related macular degeneration
AREDS	Age-Related Eye Disease Study
BCVA	Best-corrected visual acuity
CRT	Central retinal thickness
ΔBCVA	Change in best-corrected visual acuity
DM	Diabetes mellitus
ELM	External limiting membrane
ETDRS	Early Treatment Diabetic Retinopathy Study
EZ	Ellipsoid zone
HT	Hypertension
IZ	Interdigitation zone
MV	Macular volume
OCT	Optical coherence tomography
PBM	Photobiomodulation
RPE	Retinal pigment epithelium
SD-OCT	Spectral-domain optical coherence tomography
